# Optimization of Tyrosine Kinase Inhibitor-Loaded Gold Nanoparticles for Stimuli-Triggered Antileukemic Drug Release

**DOI:** 10.3390/jfb14080399

**Published:** 2023-07-27

**Authors:** Andra-Sorina Tatar, Timea Nagy-Simon, Adrian Bogdan Tigu, Ciprian Tomuleasa, Sanda Boca

**Affiliations:** 1 Interdisciplinary Research Institute in Bio-Nano-Sciences, Babes-Bolyai University, 400271 Cluj-Napoca, Romania; andra.tatar@itim-cj.ro (A.-S.T.); timea.nagy@ubbcluj.ro (T.N.-S.); 2 National Institute for Research and Development of Isotopic and Molecular Technologies, 400293 Cluj-Napoca, Romania; 3 Research Center for Advanced Medicine—MEDFUTURE, Department of Translational Medicine, Iuliu Hatieganu University of Medicine and Pharmacy Cluj-Napoca, 400347 Cluj-Napoca, Romania; adrianbogdantigu@gmail.com (A.B.T.); ciprian.tomuleasa@umfcluj.ro (C.T.); 4 Department of Hematology, Oncologic Institute Prof. Dr. Ion Chiricuta, 400015 Cluj-Napoca, Romania

**Keywords:** acute myeloid leukemia, drug delivery, nanoparticles, stimuli-responsive, tyrosine kinase inhibitors, theranostics, nanocompounds, nanomedicine

## Abstract

Tyrosine kinase inhibitor (TKI) therapy is gaining attraction in advanced cancer therapeutics due to the ubiquity of kinases in cell survival and differentiation. Great progress was made in the past years in identifying tyrosine kinases that can function as valuable molecular targets and for the entrapment of their corresponding inhibitors in delivery compounds for triggered release. Herein we present a class of drug-delivery nanocompounds based on TKI Midostaurin-loaded gold nanoparticles that have the potential to be used as theranostic agents for the targeting of the FMS-like tyrosine kinase 3 (FLT3) in acute myeloid leukemia. We optimized the nanocompounds’ formulation with loading efficiency in the 84–94% range and studied the drug release behavior in the presence of stimuli-responsive polymers. The therapeutic activity of MDS-loaded particles, superior to that of the free drug, was confirmed with toxicities depending on specific dosage ranges. No effect was observed on FLT3-negative cells or for the unloaded particles. Beyond druggability, we can track this type of nanocarrier inside biological structures as demonstrated via dark field microscopy. These properties might contribute to the facilitation of personalized drug dosage administration, critical for attaining a maximal therapeutic effect.

## 1. Introduction

Acute Myeloid Leukemia (AML) is a type of blood malignancy with thousands of new cases yearly in Europe [1,2], tens of thousands in the USA [3], and approximately 120 thousand new cases globally every year [4]. Although therapies using Tyrosine Kinase Inhibitors (TKIs) for AML have been studied more and more intensely in the last 20 years (over 3400 research papers found in the WoS database), the standard of care is still a combination of cytosine arabinoside (ara-C) with an anthracycline, since almost five decades ago [5]. However, it seems that in the near future the medical world might establish the real impact of TKI-based AML therapy, since there are already many ongoing clinical trials focused on this aspect [6]. Nevertheless, due to their more acceptable safety profile with less off-target side effects, TKIs hold a remarkable advantage that makes them suitable and promising for combinatorial or sequential treatment regimens together with other small molecule inhibitors or traditional “broad spectrum” chemotherapeutics [6].

Tyrosine Kinase Inhibitors are a class of therapeutic molecules capable of inhibiting the kinase activity of different tyrosine kinases [7], functional proteins having a key role in intracellular signal transduction and involved in a multitude of biological pathways concerning cell regulation, proliferation, differentiation, etc. [8,9]. FMS-like tyrosine kinase 3 (FLT3) is a tyrosine kinase receptor known as overexpressed in 80% of AML cases, and mutated in 30% [10]. Thus, it is a highly significant target for TKI-based AML therapy and multiple TKIs have been investigated for this purpose, such as Sunitinib, Sorafenib, Lestaurtinib, Midostaurin, Crenolanib, and Quizartinib [11,12,13]. Among these, Midostaurin (MDS) was FDA approved in 2017 for newly diagnosed AML patients [14] and has shown improvement in overall survival and event-free survival of patients if used in combination with standard chemotherapy, without increasing the incidence of severe adverse events [15,16]. Regardless, TKIs are hydrophobic molecules with low bioavailability, and chemotherapeutics are eminently toxic and induce unwanted side-effects. Considering these, efforts are still required in order to reduce their impact onto the quality of life of the patients, via approaches that can improve drug loading, targeting, focused release, and thus the overall clinical outcome.

Nanotechnology can offer certain improvements in these regards, in the form of nano-pharmacology [17,18], by combining well-known and studied therapeutic drugs with nanoparticles (NPs) and the new and compelling properties rendered by them. The high surface-to-volume ratio of NPs makes them convenient drug carriers with a high capacity for loading cargo, and their chemistry allows ease of further functionalization with molecules such as polymers. Furthermore, based on the chemical properties of the polymeric shell a multitude of advantages arise, such as the possibility for grafting targeting moieties, the ability to encapsulate therapeutic molecules or contrast agents of various chemistry as payload, and the wherewithal for controlled release of the payload at the target site based on slight endogenous differences such as pH, ionic strength, temperature, or the concentration of other chemical components [19,20]. Moreover, the composition of the NP core offers some exceptional physical and optical properties to the compound such as light-scattering and field-enhancement effects in the case of noble-metal nanoparticles or heat-releasing capabilities in the magnetic ones [21]. Multiple applications can arise from these properties among which contrast agents for imaging or tools for theranostics purposes are the most notable [22,23,24].

Stimuli-responsive polymers can aid greatly in the aspect of reducing some of the major limitations of chemotherapeutics such as ubiquitous biodistribution, non-specificity, and subsequent systemic toxicity, as they can ensure bioavailability and release of the therapeutic molecules chiefly at the diseased site, allowing lowering of the dose and dosage frequency while maintaining targeted toxicity [20,25]. There are two main strategies for pH-sensitive drug release: one is based on acid-labile linkers between the drug and the rest of the particle allowing the prodrug to become active after hydrolysis, and one is based on ionizable polyelectrolytes that dramatically change their conformation as a function of the environmental pH [20]. Moreover, the polymers that contain hydrophobic or amphiphilic sections can improve the solubility, bioavailability, and transportation of hydrophobic drugs such as TKIs, functioning as stealth vectors, as they can engulf the cargo in lipophilic pockets within their structure, and deliver them throughout the body with less clearance.

There are some research papers focused on pH-sensitive polymers and pH-triggered drug release, such as that published by Chen et al. [26] where algae-based carrageenan oligosaccharides (CAOs) were used as a green reducing agent for GNP synthesis and as a pH-triggered delivery system for epirubicin (EPI), with negligible release under physiological pH, in hepatic and epithelial cells. In another study, the hydrophobic poly (beta-amino ester) cores of polymeric pH-sensitive STEALTH^®^ nanoparticles containing the lipophilic Paclitaxel (PTX) undergo rapid dissolution at pH below 6.5 and drug release, showing good toxicity in Jurkat acute lymphoblastic leukemia cells compared to the free drug [27]. Also, anthracyclines such as doxorubicin (DOX) and idarubicin (IDA) were loaded, based on both hydrophobic and electrostatic interactions, and delivered to leukemic cells via oxidized phospholipid-based pH-sensitive micelles that show an accelerated drug release behavior under acidic conditions (pH 6) compared to 7.4 [28]. Moreover, DOX was co-loaded with a short chain ceramide (C6-ceramide) in a PEGylated bioactive lipids-based micelle system for a synergistic cytotoxic effect on drug-resistant leukemic cells, resulting in improved IC_50_ values and apoptosis, as a result of controlled drug release at pH values of 6 and 5 [29]. Unimolecular micelles composed of cyclodextrin-{poly(lactic acid)-poly(2-(dimethylamino) ethyl methacrylate)-poly[oligo(2-ethyl-2-oxazoline)methacrylate]}21 [CD-(PLA-PDMAEMA-PEtOxMA)21] were used for DOX encapsulation in the PDMAEMA and PLA layers, as well as in situ formation spots for GNP synthesis, in a study by Lin et al. [30], and drug release was shown to be enhanced with lowering pH. Moreover, an amphiphilic copolymer of PCL-PDMAEMA linked by a disulfide bond was used to efficiently deliver DOX to HepG2 and MCF-7 cells based on a pH-induced polymer re-conformation and the GSH reduction of the S–S bond [31]. In a recent work, hydrophobic PTX was encapsulated in a FFACD peptide-based hydrogel and its toxicity against K562 leukemia cells was confirmed. If the hydrogel was synthesized in the presence of amino-acid Arginine, it showed a highly improved PTX release at pH 6 compared to pH 7.4 [32]. Also, the pH-sensitive amphiphilic polymer poly [(1,4-butanediol)-diacrylate-β-NN-diisopropylethylenediamine]-polyethyleneimine (BDP) was synthesized by Yin et al. [33] and loaded with PTX leading to improvements in drug loading, murine breast cancer cell toxicity and mice survival. This system proved stable at tumor extracellular pH, but the intra-endo/lysosomal acidic environment induced a strong controlled drug release.

On the other hand, very few works have studied the pH-controlled release of hydrophobic drugs such as TKIs, from amphiphilic nano-pharmacological systems, and even fewer are focused on hematological malignancies, as discussed below. Sarkar et al. [34] have developed Pluronic-coated GNPs where the hydrophobic PPG functions as a reducing agent for the GNP synthesis, and an excellent carrier for the ZD6474 epidermal growth factor receptor (EGFR)- and vascular endothelial growth factor receptor (VEGFR)-specific TKI. The obtained particles show stability at physiological pH and a sustained release profile at an acidic pH of 5.2, as well as promising in vitro and in vivo anti breast-cancer results. Hong et al. [35] used lipid polymeric nanoparticles conjugated with dual pH-responsive and targeting peptides for the delivery of Afatinib, an anti-EGFR TKI, into colorectal adenocarcinoma (CRC) cells. The drug was loaded in the polylactic-co-glycolic acid (PLGA) core, which was surrounded by a layer of PEG-lipids, further modified with pH-sensitive and specific cell-penetrating peptides, showing increased drug release at pH 6.5 and modulated apoptosis, drug resistance, and metastasis. Focused on leukemias, recently, Erdagi and Yildiz [36] obtained polymeric nanoparticles with a methoxy poly (ethylene glycol) (mPEG) shell and a poly (ε-caprolactone) (PCL) hydrophobic core where Imatinib was encapsulated. They showed pH-sensitive release comparing buffer solutions of pH 7.4 and 3.5, and an improved cytotoxicity effect on K562 leukemia cells, compared to the free drug. Imatinib was also used by Cortese et al. [37] in a chitosan and PCL nanoparticle system, where a much quicker drug release was observed at pH 4 and 6 compared to 7.4, and a cytotoxic effect via apoptosis was induced in KU812 chronic myeloid leukemia cells. The same group then published research on an even more complex, pH-triggered, dual TKI delivery system [38], with a synergistic cytotoxic effect on KU812 cells. The authors developed a pH-sensitive chitosan core loaded with Nilotinib, surrounded by a PCL shell incorporated with a mixture of sodium bicarbonate and potassium tartrate (NaHCO_3_ + KC_4_H_5_O_6_) which quickly generated CO_2_ bubbles in acidic conditions, leading to the release of the Nilotinib.

In our previous work by Simon et al. [12], a pioneering study regarding the efficient loading of gold nanoparticles (GNPs) with selected FLT3 inhibitors, we showed that Pluronic-capped, Midostaurin (MDS)-conjugated GNPs might be promising anti-leukemic candidates with a high drug loading value of 80%, a drug release rate of 50% in simulated lysosomal conditions after 24 h, a clear reduction in cancer cell proliferation compared to control, and a superior anti-cancer effect compared to the free drug. Another work reported the use of a nanoparticle-complex based on different FLT3 inhibitors, namely Midostaurin, Sorafenib, Lestaurtinib, and Quizartinib, which were independently loaded onto gelatin-coated gold nanoparticles with promising results against THP1 acute myeloid leukemia cells [39]. We also reported that such nanobioconjugates are superior when compared with the drug alone, with data confirmed by state-of-the-art analyses of internalization, cell biology, gene analysis of the FLT3-IDT gene, and Western blotting to assess degradation of the FLT3 protein [5]. Herein, we further our research by studying MDS loading and controlled release behavior when conjugated onto GNPs functionalized with polymers that, in specific conditions, can have stimuli-responsive properties as is the case of Polyvinylpyrrolidone (PVP). By this, we are able to achieve and optimize the compound targetability. Indeed, the presented results demonstrate that a higher degree of drug loading was attained in comparison to the previously obtained PLU-conjugated nanoparticles, due to the hydrophobicity of the drug molecule and its propensity to shield within the PVP polymer. When studied in conditions that simulate lysosomal entrapment (acidic pH and presence of glutathione) as well as in control conditions simulating the extracellular fluids (physiological pH and ionic strength) over a time span of 24 h, the PVP-capped nanoparticles also demonstrate improved drug delivery within the target site in comparison with PLU-coated nanoparticles due to the controlled release effect, induced by the acidic pH. The cytotoxicity of the MDS-loaded GNPs was compared to that of the free drug molecule, with very effective results on the MV-4-11 (CRL-9591™) target cell line (cell viability below 15%) that carries the FLT3-ITD mutation [40,41] and little observable effect in the case of negative control, OCI-AML-3 (ACC 582) cell line. Moreover, the ability of the particles to be imaged by dark field microscopy allows the monitoring of their internalization and distribution inside cells and cellular compartments. This feature is of great importance in terms of efficient administration and targetability of the drug cargo.

## 2. Materials and Methods

### 2.1. Materials

Hydrogen tetrachloroaurate (III) trihydrate (HAuCl_4_·3H_2_O, 99.99%), Sodium citrate tribasic dihydrate ACS reagent, Citric acid ACS reagent, Pluronic F127 (PLU), Polyvinylpyrrolidone, mw 10,000 (PVP), Midostaurin hydrate (>98%) (MDS), L-Glutathione reduced ≥98.0% (GSH) were obtained from Sigma-Aldrich, Merck, St Louis, MO, USA. Paraformaledehyde was acquired from ThermoFisher Scientific, Waltham, MA, USA. The Phosphate-Buffered Saline (PBS), Dulbecco Phosphate-Buffered Saline (DPBS), Iscove’s Modified Dulbecco’s Medium (IMDM), Minimum Essential Media (MEM), Fetal Bovine Serum (FBS), Penicillin/Streptomycin and Glutamine were purchased from Gibco^®^, Grand Island, NY, USA. The XTT reagent mixed with Electron Coupling Reagent are from Invitrogen, Waltham, MA, USA. All other reagents used during the experiments are of analytical grade and were utilized without further purification. The aqueous solutions were prepared with ultrapure water (with a resistivity of 18.2 MΩ cm) from a Milli-Q purification system (Millipore, Merck KGaA, Darmstadt, Germany).

### 2.2. Methods

#### 2.2.1. Synthesis and Biofunctionalization of MDS-Loaded Gold Nanoparticles (MDS-GNP)

Gold nanoparticles were fabricated using the Turkevich–Frens synthesis protocol [42,43]. The Pluronic-conjugated MDS-loaded GNPs were fabricated using a previously developed protocol [12], the self-assembly/structurization being ensured by hydrophobic interactions between polypropylene glycol (PPG) regions of the Pluronic block-copolymer on the inside, proximally to the particle surface and able to engulf the hydrophobic MDS, and hydrophilic polyethylene glycol (PEG) interactions on the outside, in contact to the aqueous media and ensuring solubility. Otherwise, PVP is uniformly amphiphilic and can load the hydrophobic TKI throughout, being also able to adsorb at the nanoparticle surface electrostatically, presumably through H-bonds between its C=O and the carboxyl groups of citrate molecules capping the synthesized GNPs [44,45]. For drug loading, 40 µL of 1 mg/mL MDS were added to the colloidal solution followed by the rapid addition of the polymer at different concentrations depending on the polymer type and its stealth characteristics: 2 mM Pluronic to obtain the final product denoted GNP-MDS-PLU and 1 mM PVP for the preparation of GNP-MDS-PVP nanoparticles, respectively, which were incubated for 1 h and 2 h, respectively, before purification by centrifugation (16 k rpm-25 min, and 15 k rpm-25 min, respectively), followed by resuspension in the desired buffer solution. Based on previously obtained calibration curves (SI Figure S1), we used the value of MDS characteristic absorbance measured in various configurations for quantification and calculation of the drug loading efficiency (LE%).

#### 2.2.2. Evaluation of the Drug Release Profile

After centrifugation for the removal of the excess MDS and polymers, the particles were resuspended in different types of buffer solutions: (i) acid citrate buffer (pH 4.5); (ii) acid citrate buffer (pH 4.5) with a GSH concentration of 10 mM; and (iii) PBS (pH 7.4) and incubated at 37 °C for 1, 3, 8 and 24 h, respectively. The acid buffers were used in order to mimic the lysosomal environment, and the PBS is used as a control, and also due to its ionic resemblance to the extracellular fluids. At the selected time checkpoints, the samples were centrifuged at 37 °C to separate the nanoparticles from the supernatant which contains the released drug molecules. The absorbance spectra of the two fractions were measured to quantify the concentration of the released drug and that of the drug remaining within the nanoparticle structure, calculations based on previously obtained calibration curves (SI Figure S1). MDS quantification was based on area under the curve measurements in the 300–380 nm region.

#### 2.2.3. Particle Characterization

UV-Vis-NIR extinction spectra were acquired using a Jasco V-670 UV−Vis−NIR spectrometer at 1 nm spectral resolution, in 2 mm path length quartz cuvettes. Particle size distribution (via Dynamic Light Scattering) and zeta-potential were measured at 25 °C using the Zetasizer NanoZS90 from Malvern Panalytical Ltd., Malvern, UK. Particle morphology was determined by Transmission Electron Microscopy using a JEOL model JEM1010 microscope, JEOL Ltd., Tokyo, Japan.

#### 2.2.4. Cell Culture Protocol

The FLT3-ITD mutated AML cell line MV-4-11 (CRL-9591™) and the wild-type AML cell line OCI-AML-3 (ACC 582) were cultured in sterile cell culture flasks, at 37 °C and 5% CO_2_, in a humidified chamber. IMDM cell culture medium, supplemented with 10% Fetal Bovine Serum (FBS), 1% Penicillin/Streptomycin, and 1% Glutamine was used for maintaining MV-4-11 in optimal growth conditions, while for OCI-AML-3 MEM cell culture medium supplemented with 20% FBS, 1% Penicillin/Streptomycin, and 1% Glutamine was used.

#### 2.2.5. XTT Assay

XTT assay was performed using the CyQUANT XTT Cell Viability Assay (Invitrogen, Carlsbad, CA, USA) with a seeding density of 15 × 10^3^ cells/well in 100 μL of cell culture medium. After 24 h incubation, different concentrations of GNP-MDS-polymer, GNP-polymer, free MDS and free DMSO were added to the cell samples. The cells were incubated in the presence of the particles for 24 h; thereafter, the XTT reagent mixed with Electron Coupling Reagent was added to each well and incubated for 4 h at 37 °C, protected from light. The cell viability was measured at 450 nm and 660 nm using a TECAN SPARK 10 M spectrophotometer (TECAN, Austria GmbH, Grodig, Austria). Data analysis and graphical representation were performed using Graph Pad Prism 8, and the results were expressed as mean standard deviation (GraphPad Software, San Diego, CA, USA).

#### 2.2.6. Cell Preparation for Dark Field Microscopy

15 × 10^5^ cells were seeded in sterile 24-well plates in 1 mL of cell culture medium at 37 °C and 5% CO_2_. After 24 h of incubation the cells were treated with GNP-MDS-polymer, GNP-polymer, free MDS, and DMSO at a final concentration of 10% in culture medium, bringing the cells at a final volume of 2 mL of culture medium mixed with Dulbecco Phosphate Buffered Saline—DPBS 1X and incubated for 24 h. After the treatment, the cells were removed from the 24-well plate and washed three times with DPBS 1X then fixed with 4% paraformaldehyde solution for 10 min followed by three washing steps with DPBS 1X solution, and then stored at 4 °C in 500 μL of DPBS 1X.

#### 2.2.7. Microscopic Analysis

To investigate the cell density in each well after treatment, the plates were analyzed using an inverted Zeiss Axio Observer Z1 microscope (Zeiss, Jena, Germany). Bright field and dark field (DF) microscopy were performed on cells immobilized onto Ibidi 30 μ-Dishes-50 mm (ibidi GmbH, Gräfelfing, Germany). A 100 W halogen lamp was used for sample illumination with constant intensity on each sample and focused through a high numerical immersion condenser (NA = 1.4). The scattered light was collected by a Plan-Apochromat oil immersion 63× objective (NA= 0.7−1.4). Images were acquired using an AxioCam Icc Rev.4 CCD camera (1.4 megapixels, Zeiss) with the same integration time for each sample and processed by the ZEN software.

## 3. Results and Discussion

### 3.1. Fabrication and Characterization of Drug-Loaded Gold Nanoparticles

**Figure 1:**
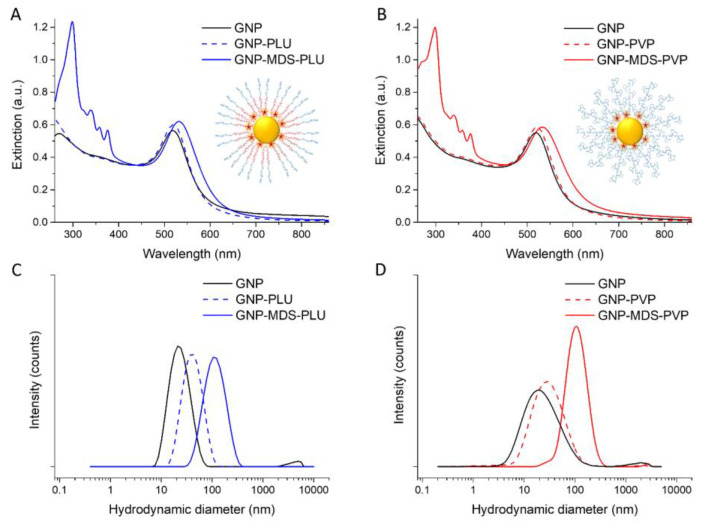
UV-Vis-NIR extinction spectra of (**A**) Pluronic-nanoconjugates and (**B**) PVP-nanoconjugates. Schematic structural representations as insets. DLS hydrodynamic diameter data of (**C**) Pluronic-nanoconjugates and (**D**) PVP-nanoconjugates. Black lines: pristine GNPs; blue lines: Pluronic-conjugated particles; red lines: PVP-conjugated particles; dotted line: GNP-polymers; full colored lines: GNP-MDS-polymers.

Spherical gold nanoparticles (morphological characterization by TEM micrograph can be seen in SI Figure S2) were purified and used for further functionalization. Figure 1A presents the UV-Vis-NIR extinction spectra of the particles before functionalization (black line) and of the drug-loaded Pluronic-coated nanoconjugate product after purification and resuspension in PBS, respectively (blue line). Polymer-conjugated and nonconjugated GNPs (blue dotted line) are also illustrated for comparison. The MDS characteristic peaks are visible in the 300–380 nm region, while drug and polymer adsorption at the particle surface are demonstrated by the red-shift of the localized surface plasmon resonance (LSPR) band position. For these particles, we calculated a loading efficiency of 84.7%. It is worth mentioning that particle incubation with the MDS drug without the addition of a stabilizing polymer leads to a rapid particle aggregation after 1–2 min up to 30 min interaction, as shown in SI Figure S3.

To optimize the GNP-MDS-PVP nanoconjugates, we tested the functionalization efficiency for two different PVP polymer concentrations (1 mM and 4 mM). The results (SI Figure S4) showed identical behaviors of the samples, confirming that 1 mM PVP is the required concentration for an efficient polymeric coating. Further optimization was performed regarding particle purification via centrifugation, to ensure a correct separation of the supernatant and a preservation of the nanoconjugate integrity, hence the selection of 15,000 rpm for complete particle purification (data not shown). Thirdly, we explored the MDS loading efficiency of the system for two different protocol configurations: (i) incubation of the drug with the GNPs followed by the addition of the polymer, and (ii) mixture of the MDS with the PVP solution, followed by their addition to the GNPs solution. The results showed a better degree of drug loading in configuration (i), and higher MDS loss as remaining in the supernatant after washing the sample in configuration (ii) (SI Figure S5).

The presence of the drug specific absorption bands in the 300–380 nm region and the red-shift of the LSPR band position of the nanoparticles in the UV-Vis-NIR extinction spectra of the GNP-MDS-PVP nanoconjugates (Figure 1B) confirm the efficient drug loading. The UV-Vis-NIR extinction spectra of the particles before functionalization (black line), the polymer conjugated, unloaded GNPs (red dotted line), and the final drug-loaded nanoconjugate product after purification and resuspension in PBS (red line) are presented for comparison. The PVP polymer not only provides “smart” functionality via its stimuli-sensitive properties, but also renders the nanoparticles biocompatible and stable, since no modification of the spectra is observed when particles were measured at different time points (SI Figure S6). For these particles, we calculated a loading efficiency of 97.7%. Such high drug loading yield can be explained by the hydrophobicity of the TKI molecule, a property which ensures an enhanced incorporation in the polymeric structure. Similar results, with calculated encapsulation efficiencies above 90% were previously obtained for multiple TKIs [34,46]. Moreover, to ascertain the reproducibility of the preparation protocol, we have measured the extinction spectra of nanoconjugates prepared at different time points, and the results, indicating highly reproducible samples, are presented in the Supplementary Information (SI Figure S6).

Both types of nanoconjugates have a very high MDS loading efficiency (between 84% and 94%), being facilitated by the hydrophobic nature of the TKI drug molecules. The LSPR position is red-shifted after the functionalization of the GNPs from 518 nm to 527 and 533 nm, respectively, confirming the adsorption of the drug and polymer molecules onto the surface of the particles. In the absence of the drug, the polymers alone lead to smaller shifts, of 1 nm the case of PLU and 3 nm for PVP. This is explained by the higher refractive index (*n*) values of the polymers compared to that of the water: 1.46 for PLU and 1.55 for PVP. DLS measurements corroborate the results confirming the successful functionalization by the increase in hydrodynamic diameter of the particles after drug and polymer adsorption from 24.94 ± 3.8 nm for bare nanoparticles to 122.96 ± 13.6 nm and 231.67 ± 23 nm, respectively. Coating of the GNPs with only polymers leads to an increase in their hydrodynamic diameter but to a lesser extent (to 44.28 ± 0.29 nm and 73.56 ± 6.6 nm for PLU and PVP, respectively). The zeta-potential measurements show a value of −61.6 ± 1.1 mV for the pristine GNPs and −2.35 ± 0.9 mV after the Pluronic shielding. Both types of polymers modify the zeta-potential of the particles [47] to values approaching neutral charge: GNP-MDS-PLU particles have a zeta-potential of −0.99 ± 0.2 mV, and PVP-coated GNPs have values around −5.07 ± 0.6 mV (without drug) and −5.29 ± 1.6 mV (with MDS), also conferring steric stability to the nanocomplexes (Table 1).

### 3.2. Evaluation of the Drug Release Profile

**Figure 2:**
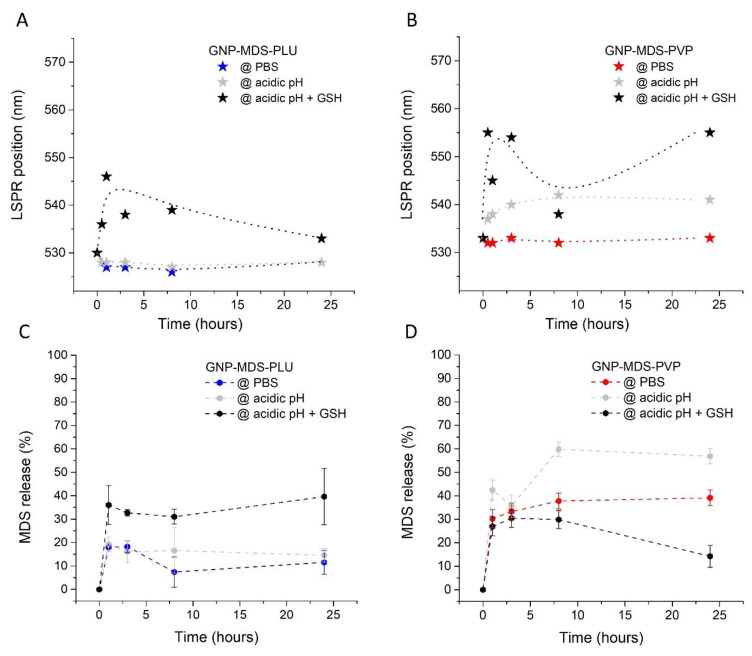
Time-dependent LSPR band positions of (**A**) GNP-MDS-PLU and (**B**) GNP-MDS-PVP measured in various incubation media. MDS drug release rates from (**C**) GNP-MDS-PLU and (**D**) GNP-MDS-PVP.

As the nanocomplexes were subjected to the release conditions, we also tracked the position of the GNPs characteristic LSPR band during the 24 h of the test. These details are represented in Figure 2A,B, and show a burst destabilization of the GNP-MDS-polymer nanocomplexes in the first 1–3 h, succeeded by a slight re-stabilization in the following hours. Still, in each of the experimental configuration, the polymers behaved slightly differently and in concordance to their physico-chemical properties, and the release buffer media. Pluronic, as a non-pH-responsive polymer, was used as control and has proven to be the most stable particle coating in the PBS and acidic pH release conditions, while PVP is more responsive and showing a more substantial response in the LSPR band shift. As regarding the buffers, PBS was used as a control and has shown the lowest influence on particle stability throughout the experiments. The acidic conditions imparted clearer destabilization effects onto the PVP polymer, followed by the GSH buffer, where presence of the GSH molecules leads to displacement of the stabilizing polymers from the particle surface leading to the strongest LSPR shift (i.e., particle aggregation effect) observable for both polymers.

To calculate drug release rates, we explored two different calculation methods based on the area under the curve of the four MDS peaks in the 300–380 nm region in the extinction spectra, which are discussed below. On the one hand, we used a priori obtained calibration curves (SI Figure S1) to quantify the amount of MDS released from the nanostructures into the three different release media at pre-selected time-points. This value was used to calculate the drug release rate (%) compared to the concentration initially loaded on the particles which represents 100% (data not shown). This method has the advantage of being straightforward with calculating the amount of released drug, but also the disadvantage of quantifying the MDS in three different media (i.e., each of the buffers used), which are furthermore not optimal solvents for the free hydrophobic MDS molecules that tend to precipitate. On the other hand, after the particles were incubated with the release media for the pre-selected time-frames and they were separated from the released drug molecules, we measured the extinction spectra of the particles resuspended in the isotonic buffer PBS, which we used to quantify the remaining MDS, thus allowing the calculation of the amount of released drug in each buffer and release conditions, as seen in Figure 2C,D. Although this method is an indirect way to calculate the degree of drug release, it proved more robust, since all the analyzed spectra are measured in PBS and the relevant quantities of drug are encompassed in the more MDS-solving microenvironment of the nanostructures.

Thus, the results regarding drug release presented in Figure 2C show that the GNP-MDS-PLU particles are most stable in PBS, closely resembled by their behavior in acid pH, with release rates of approximately 13% and 25% after 8 h, respectively, both buffers inducing a 16–17% release rate after 24 h. This is corroborated with a mostly constant LSPR band position throughout the 24 h experiment, showing stability of the polymeric coverage alongside a relatively low level of diffusion of the MDS molecules. By contrast, due to the thiol functional group, GSH binds preferentially to the surface of the particles, displacing both the adsorbed therapeutic and the polymeric molecules. Therefore, a higher content of MDS is released alongside Pluronic molecules, probably structured as micelles containing MDS. After 24 h, release rate was calculated as 50%, a result similar to that previously shown in our paper by Simon et al. [12]. On the other hand, the GNP-MDS-PVP particles are overall more responsive in regard to drug release rates (Figure 2D). For this case, the LSPR band position remains mostly constant throughout the experiment even in the control buffer (PBS), the polymer not being destabilized, and the release rates reaching less than 30% after 3 h of incubation. As PVP is a pH-sensitive polymer, the acidic pH buffer induces a burst release effect of around 45% in the first 3 h, leading to values of approximately 60% after more than 8 h of incubation, alongside a moderate LSPR shift due to structural changes of the polymer under the influence of the acidity. An interesting release behavior occurs in the presence of GSH: although it is the most destabilizing agent, and hence induces the largest LSPR band shift, it leads to the lowest release rates of the drug. We infer that this is a result of a combination of factors: (i) the drug is being trapped alongside the GSH molecules covalently bound to the gold surface, and (ii) PVP has a lower capacity to engulf the hydrophobic MDS molecule in such acidic conditions. Moreover, the small size of GSH compared to the much higher molecular weight PVP polymer results in a thinner organic corona layer at the particle surface which decreases the steric hindrance stability and hence favors the aggregation of the nanoparticles, which corroborates with the LSPR band shift.

### 3.3. Assessment of Particle Internalization by Dark Field Microscopy Imaging

**Figure 3:**
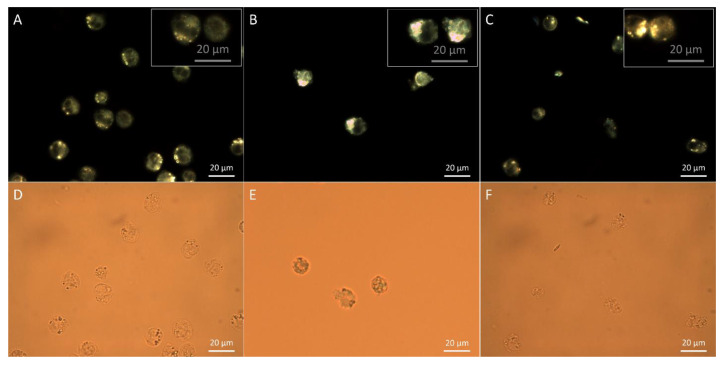
LSPR Dark field and bright field microscopic images of MV4-11 cells incubated in the presence of GNP-MDS-PLU (**B**,**E**) and GNP-MDS-PVP (**C**,**F**) nanocompounds. Corresponding images of control, free of particle samples (**A**,**D**).

To ascertain the nanoparticle internalization by the targeted cells we imaged the cellular samples under dark field microscopy configuration. Figure 3B,C presents the dark field microscopy images of MV4-11 cells after being incubated with GNP-MDS-PLU and GNP-MDS-PVP bioconjugates. The nanoparticles can be tracked inside cells as sparkling yellow-to-orange dots due to the light scattering at their plasmon resonance wavelength. In the samples incubated in the presence of the nanoparticles, a variation in the size of the luminescent dots is observable, a feature related to the particle agglomeration inside cellular vesicles and their light-scattering cross sections for specific frequencies that are much larger than their actual cross-sectional areas. The luminescent spots observable in the control, free of particle, sample are easily distinguished from the particle-generated ones as they present lower intensity and no color variation, being attributed to the light scattering of endosomal structures [48] (Figure 3A).

### 3.4. Cellular Toxicity

**Figure 4:**
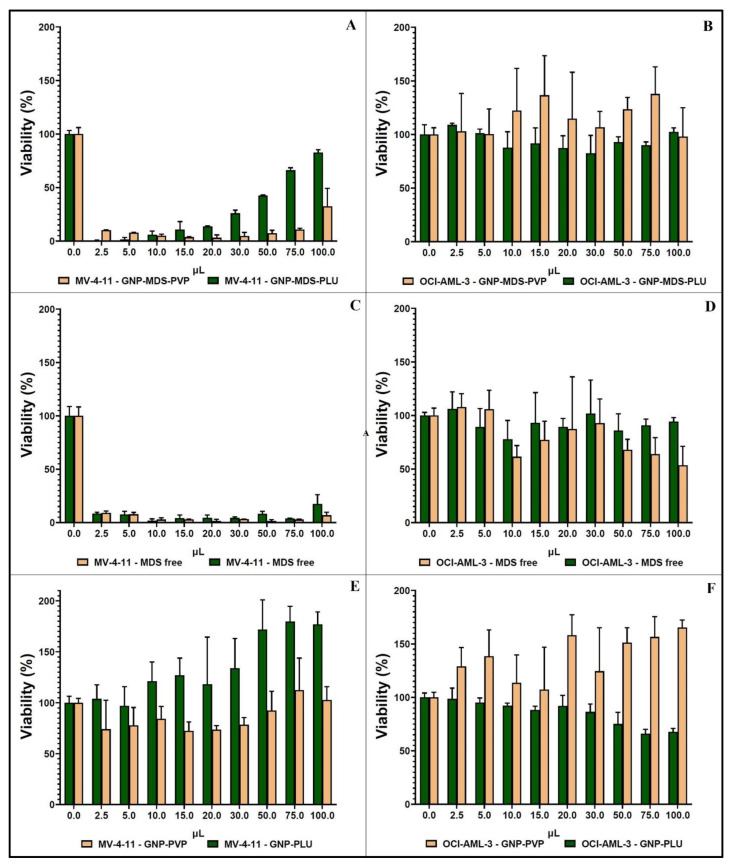
Histograms showing XTT results of the cell viability tests on MV4-11 cells (left column) and OCI-AML3 cells (right column) incubated with: GNP-MDS-polymers (**A**,**B**); free MDS (**C**,**D**); and GNP-polymers (**E**,**F**).

The cellular toxicity of the two types of GNP-MDS-polymer nanocomplexes was investigated using XTT assay, and the results are presented in Figure 4. The percentage of live cells treated with various concentrations of nanocomplexes is compared to an untreated, control sample. The left column contains results obtained on the FLT3-positive cell line, MV-4-11, and the right column shows data obtained on the FLT3-negative, OCI-AML3 cell line, used as control. It is visible from the graphics that the GNP-MDS-polymer nanoparticles have a significant cytotoxic effect on the MV4-11 cell line (Figure 4A), as do equivalent concentrations of the free drug, used as control (Figure 4C). By contrast, GNPs-polymer nanoparticles without the therapeutic drug show no cytotoxicity (Figure 4E), which confirms that the MDS cargo from the GNP-MDS-polymer compounds is responsible for the cytotoxic effect. On the other hand, none of the cell viability values calculated for the FLT3-negative cell line varies much from 100% (Figure 4B,D,F), demonstrating that this modality of treatment is targeted to the FLT3-carying cells exclusively, which is an important aspect in improving the specificity of the therapeutic process and lessening overall unwanted side-effects. Pointedly, it can be observed that GNP-MDS-PLU shows an apparent increase in cell viability at high particle concentrations, which seems counterintuitive, as higher nanopharmaceuticals concentrations lead to lower cell viability. This result is attributable as a false positive and is caused by the contribution of the optical absorption of GNPs in the highest concentrations used herein, which overlaps the wavelength range used for the quantification of the formazan molecule in the XTT viability assay (450 nm and 660 nm). This effect is increasingly more visible at higher particle concentrations in the case of both GNP-PLU particles (viability values higher than 100%, up to 180%), and GNP-MDS-PLU (an additional 80% viability value). However, this false-positive effect is not observed for the PVP-coated particles for which, due to the pH-reactivity of the polymer, the exposure to intracellular conditions leads to higher particle aggregation and to a shift in the LSPR position, as seen in Figure 2B, which will no longer interfere with the absorbance of the formazan. The explanations above are equally supported by the microscopic images of the MV-4-11 cells incubated in the presence of the GNP-MDS-PLU, which illustrate a substantial decrease in cell population as the concentration of the particle-drug polymeric compound increases (SI Figure S7). Not least, the entirety of the toxic effect observed in samples incubated with the free MDS is solely attributed to the therapeutic molecule, as another set of control samples of cells incubated with free DMSO, the solvent of the MDS stock solution, showed no cytotoxic effect (SI Figure S6).

### 3.5. The FLT3 Signaling Pathway as a Pharmacological Target: Mechanisms of Drug and Drug-Nanoparticle Compounds Action

FLT3 protein is a transmembrane receptor composed of an extracellular domain for ligand binding, a transmembrane region, an internal juxtamembrane domain, and two cytosolic kinase domains. Pairs of these proteins form and self-phosphorylate in the presence of the specific FLT3 ligand (FL), which is a small 235 amino-acid molecular factor expressed by most tissues such as spleen, thymus, bone marrow, peripheral blood (specially mononuclear cells), as well as prostate, testis, ovaries, placenta, kidneys, colon, small intestine, lung, and heart [49]. By contrast to the ubiquity of FL, the FLT3 receptor is mainly present in early progenitor cells of hematopoietic lineages [50]. In healthy tissues, the phosphorylated cytoplasmic domain of FLT3 physically associates and further phosphorylates a number of downstream proteins such as the p85 subunit of phosphoinositol-3-kinase (PI3K), phospholipase C, growth factor receptor bound protein (Grb2), the Ras GTPase, or the Src family of kinases. Further phosphorylations downstream, the mitogen-activated protein kinase (MAPK) pathway and the PI3K/protein kinase B (Akt) are activated [50,51,52]. The oncogenic mutations that lead to constitutive activation of FLT3 result in activation of Ras/MAPK kinase and PI3K/Akt-mTOR pathways as downstream proliferative signaling pathways and in a loss of the auto-inhibitory functions as well [50]. More, the FLT3-ITD mutated form can also activate the STAT5 pathway and subsequent cell growth factors like cyclin D1, c-myc, and the anti-apoptotic gene p21 [53]. Additionally, there is evidence that the FLT3-ITD mutation induces increased reactive oxygen species (ROS) production, probably via the STAT5-induced RAC1 activation which is involved in certain ROS-producing oxidases. Thus, this mutation’s aggressiveness could be a result of a cycle of genomic instability driven by increased DNA damage and repair errors [54]. The Internal Tandem Duplication (ITD) form of the FLT3 (FLT3-ITD) appears due to a mutation in exon 14 of the FLT3 gene, on chromosome 13 (13q12), where a section of three to more than 400 base-pairs can get duplicated in-frame (as multiples of three base-pairs, thus maintaining the functionality of the downstream phosphorylating protein domains). This leads to the formation of FLT3 receptor proteins with larger/longer juxtamembrane regions that result in the disruption of the proteins’ autoinhibitory function and a constitutive autophosphorylation [40,41].

Midostaurin is a first-generation multi-targeted TKI with a wide range of action: it, together with its major active metabolites (CGP62221; CGP52421 [55]), can inhibit the phosphorylative activity of wild-type and/or mutant FLT3 (including the FLT3-ITD form present in the MV4-11 model cell line used herein), protein kinase C, KIT, VEGFR, and PDGFR [56]. MDS belongs to the adenosine triphosphate (ATP)-competitive type-I class of kinase inhibitors, as it binds to the Asp-Phe-Gly (DFG) motif of the kinases in the catalytically active “DFG-in” conformation and orthosterically competes with ATP [57]. It demonstrates, however, tenfold lower inhibition of the wild-type FLT3 compared to mutant forms, which is an important advantage in reducing non-wanted side-effects [58].

**Figure 5:**
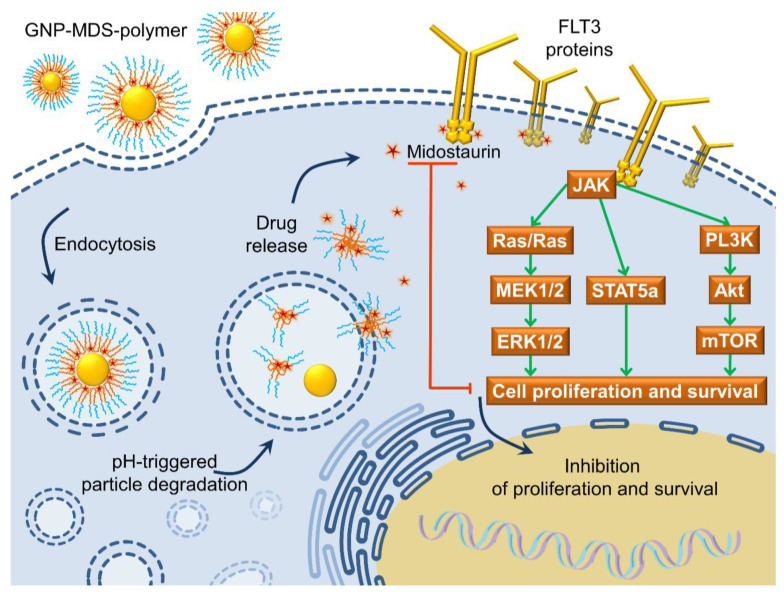
Schematic representation of the internalization process of a GNP-MDS-polymer nanocompound by a FLT3-positive cell, followed by pH-triggered particle degradation leading to the release of the drug.

The results presented in the current paper suggest a cytotoxic mechanism of action of the MDS-loaded nanoparticles which is based on a triggered drug release taking place in the endo-lysosomal system (Figure 5) due to the physico-chemical conditions within this particular cellular compartment (lowering pH down to 4.5, and a 10 mM glutathione presence). The pH-sensitive polymer coating of the GNPs changes structural conformation, while the GSH molecules chemically adsorb to the GNPs surface displacing the weaker bound MDS. The therapeutic molecules escape the endosomal vesicles and can interact with the intracellular domain of the FLT3 proteins inhibiting auto-phosphorylation and the downstream signaling cascades discussed above [56,59].

## 4. Conclusions

In this work, we developed a class of drug-delivery nanocompounds that can increase the targeting efficiency of tyrosine kinase inhibitor drugs, currently used in the treatment protocols of blood cancers. By loading the TKI Midostaurin onto various types of polymer-coated gold nanoparticles (herein Pluronic and Polyvinylpyrrolidone) we were able to fabricate nanocompounds that can simultaneuously track and neutralise the FMS-like tyrosine kinase 3 (FLT3) in MV4-11 AML cells, without influencing the viability of the control FLT3-negative cells, which is a prerequisite for effective drug clearence. Drug release dynamics were investigated at parameters that mimic the cancer cell physiological conditions and the results show particle destabilisation under specific stimuli, and hence improved drug release. Beyond the therapeutic effect, the proposed nanocompounds show imaging capabilities inside cells as they strongly scatter light when visualized under a dark field microscopy configuration, which indicate a theranostic potential. TKI-based therapy for cancer therapeutics is a growing direction in pharmaceutical development, and a combined administration with rapid and dynamic microscopic tracking of the drug compounds might contribute to the facilitation of personalized drug dosage administration, critical for attaining a maximal therapeutic effect with less side effects.

## Figures and Tables

**Table 1:** Characterization of GNPs and functionalization parameters: hydrodynamic diameters, polydispersity indices, zeta-potential values, LSPR band positions, drug loading efficiencies, and drug final concentrations.

	HDD (nm)	PDI	Zeta-Potential (mV)	LSPR (nm)	LE (%)	MDS Concentration (µg/mL)
**GNP**	24.94 ± 3.8	0.525	−61.6 ± 1.1	518	-	-
**GNP-PLU**	44.28 ± 0.29	0.716	−2.35 ± 0.9	519	-	-
**GNP-MDS-PLU**	122.96 ±13.6	0.482	−0.99 ± 0.2	527	84.7	27.12
**GNP-PVP**	73.56 ±6.6	0.603	−5.07 ± 0.6	521	-	-
**GNP-MDS-PVP**	231.67 ±23	0.247	−5.29 ± 1.6	533	93.6	30.19

HDD: hydrodynamic diameter; PDI: polydispersity index; LSPR: localized surface plasmon resonance; LE: loading efficiency; MDS: Midostaurin; GNP: gold nanoparticles; GNP-PLU: Pluronic-coated GNPs; GNP-MDS-PLU: MDS-loaded, PLU-coated GNPs; GNP-PVP: polyvinylpyrrolidone-coated GNPs; GNP-MDS-PVP: MDS-loaded, PVP-coated GNPs.

## Data Availability

The data presented in this study are available on request from the corresponding author.

## Supplementary Materials

The following supporting information can be downloaded at: https://www.mdpi.com/article/10.3390/jfb14080399/s1, Figure S1: Calibration curves for MDS quantification; Figure S2: TEM micrograph of GNPs; Figure S3: Timelapse of extinction spectra of GNPs incubated with MDS; Figure S4: Extinction spectra of GNPs incubated with different PVP concentrations; Figure S5: Extinction spectra of GNPs functionalized with MDS and PVP in different protocol configurations; Figure S6: XTT assay of MV4-11 and OCI-AML3 cells incubated with DMSO. Figure S7: Histograms showing XTT assessment of cell viability of MV4-11 cells (left) and OCI-AML3 cells (right) incubated with DMSO, as control; Figure S8: Bright field images of MV4-11 cells incubated for 24 hours with different GNP-MDS-PLU volumes: (A) control, 0 µL; (B) 2.5 µL; (C) 5 µL; (D) 10 µL; (E) 15 µL; (F) 20 µL; (G) 30 µL; (H) 50 µL; (I) 75 µL; (J) 100 µL.
