# Supplementary material for: Optimization of Tyrosine Kinase Inhibitor-Loaded Gold Nanoparticles for Stimuli-Triggered Antileukemic Drug Release
Source: J Funct Biomater. 2023 Jul 27;14(8):399. doi: 10.3390/jfb14080399 (PMC10455807; doi:10.3390/jfb14080399)
Supplement: Supplementary file 1 [file jfb-14-00399-s001.zip › jfb-2515642-supplementary.pdf]

Optimization of tyrosine kinase inhibitor-loaded gold nanoparticles for stimuli-triggered antileukemic drug release

Andra-Sorina Tatar[1,2], Timea Nagy-Simon[1], Adrian Bogdan Tigu[3], Ciprian Tomuleasa[3,4], Sanda Boca[1,2] *

[1]Interdisciplinary Research Institute in Bio-Nano-Sciences, Babes-Bolyai University, 400271 Cluj-Napoca, Romania

[2]National Institute for Research and Development of Isotopic and Molecular Technologies, 400293 Cluj-Napoca, Romania

[3]Research Center for Advanced Medicine - MEDFUTURE, Department of Translational Medicine, Iuliu Hatieganu University of Medicine and Pharmacy Cluj-Napoca, 400347 Cluj-Napoca, Romania

[4]Department of Hematology, Oncologic Institute Prof. Dr. Ion Chiricuta, 400015 Cluj-Napoca, Romania

*Correspondence: sanda.boca@ubbcluj.ro

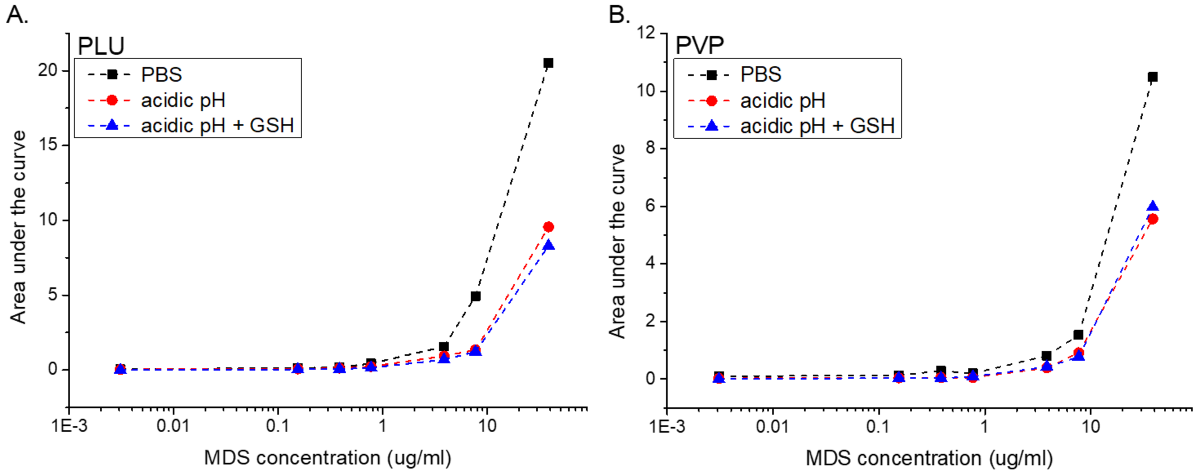

Figure S1. Calibration curves for MDS quantification, obtained for each of the polymers (A: Pluronic B: PVP) in different release media solutions (black: PBS; red: acidic pH; blue: GSH in acidic pH).

For each type of polymer and buffer solution, we constructed a separate calibration curve, to account more closely the optical effects that indicate the presence of the excess polymer molecules with the released MDS and their behavior in different release media. For the calibration curves we used *simulated supernatant solutions* obtained from identical particle functionalization protocols (GNPs + polymers), in the absence of MDS, and thus the solutions contained similar amounts of traces of washed off polymer. Particularly, we constructed 6 calibration curves based on the *area under the curve* measurements (from the four peaks in the 300-380 nm region) as a function of *MDS concentration*. By fitting, 6 trendline equations were obtained, and the data was used to extrapolate MDS concentration in the samples, both for the calculation of *Loading Efficiency (%)* and for tracking the MDS controlled release.

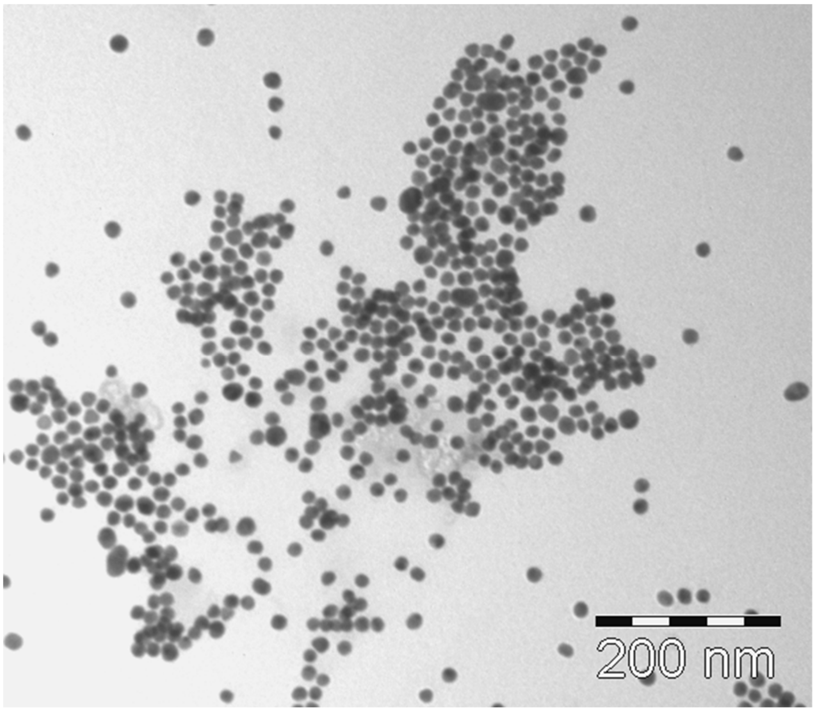

Figure S2. TEM micrograph of the GNPs. Scale is 200 nm

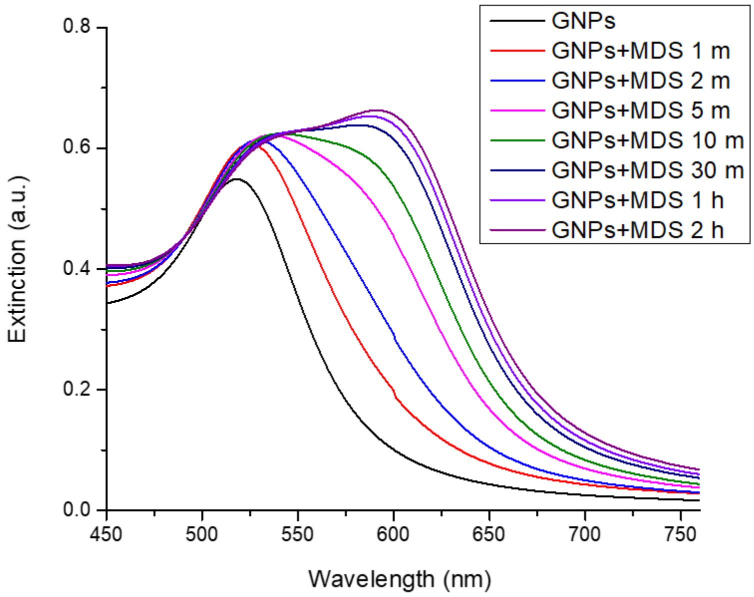

Figure S3. Extinction spectra of GNPs incubated with MDS solely. Time-lapse showing aggregation-inducing effect of MDS up to 2 hours.

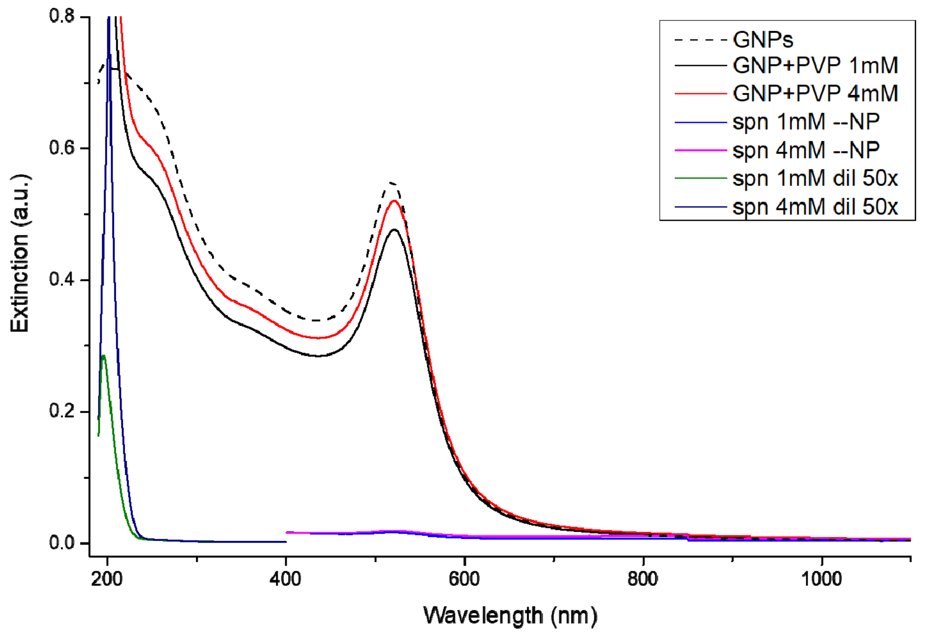

Figure S4. Extinction spectra of GNPs incubated with different PVP concentrations: 1 mM vs 4 mM.

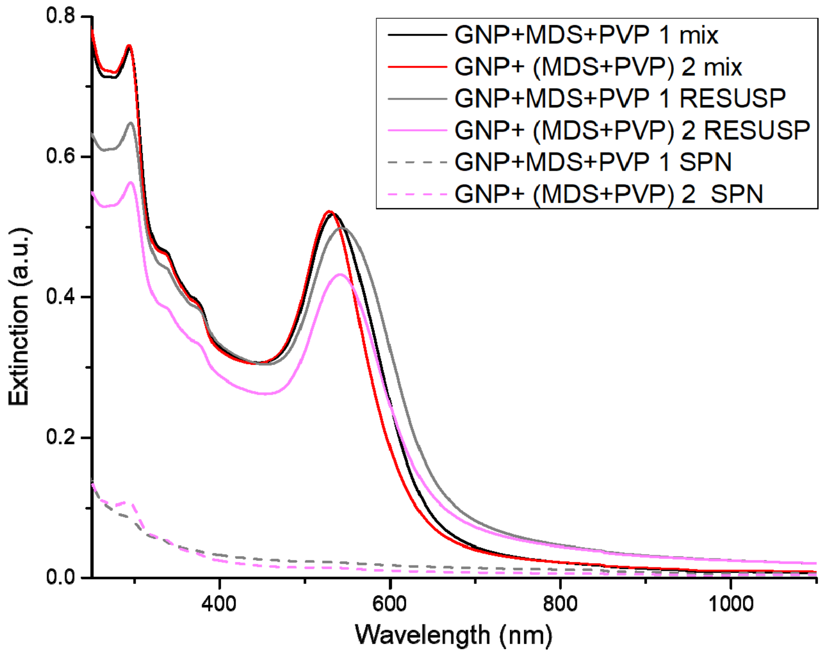

Figure S5. Extinction spectra of GNPs conjugated with MDS and PVP in different protocol configurations: ((GNP + MDS) + PVP): addition of MDS before polymer introduction; (GNP + (MDS+PVP)): pre-incubation of the MDS with the PVP before addition to the GNPs.

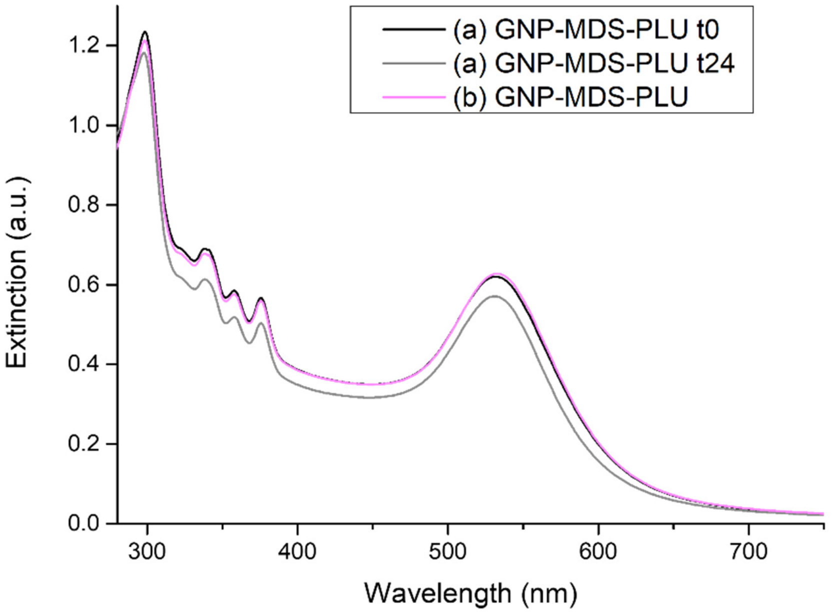

Figure S6. Extinction spectra of GNPs-MDS-PLU nanoconjugates: sample (a) measured at different time points (t0 -as prepared; t24 -24h storage), and sample (b) prepared by the same protocol.

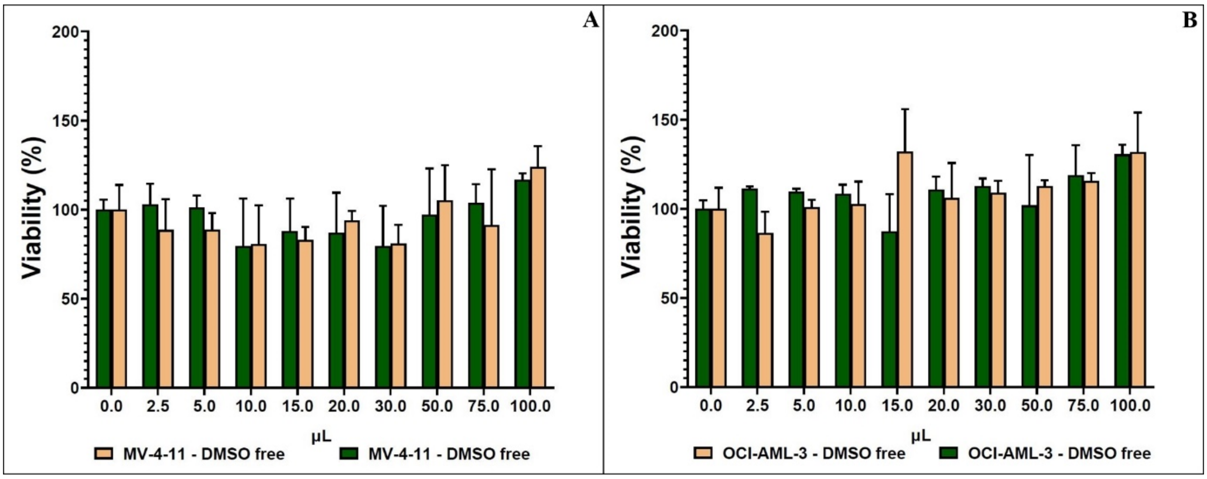

Figure S7. Histograms showing XTT assessment of cell viability of MV4-11 cells (left) and OCI-AML3 cells (right) incubated with DMSO, as control.

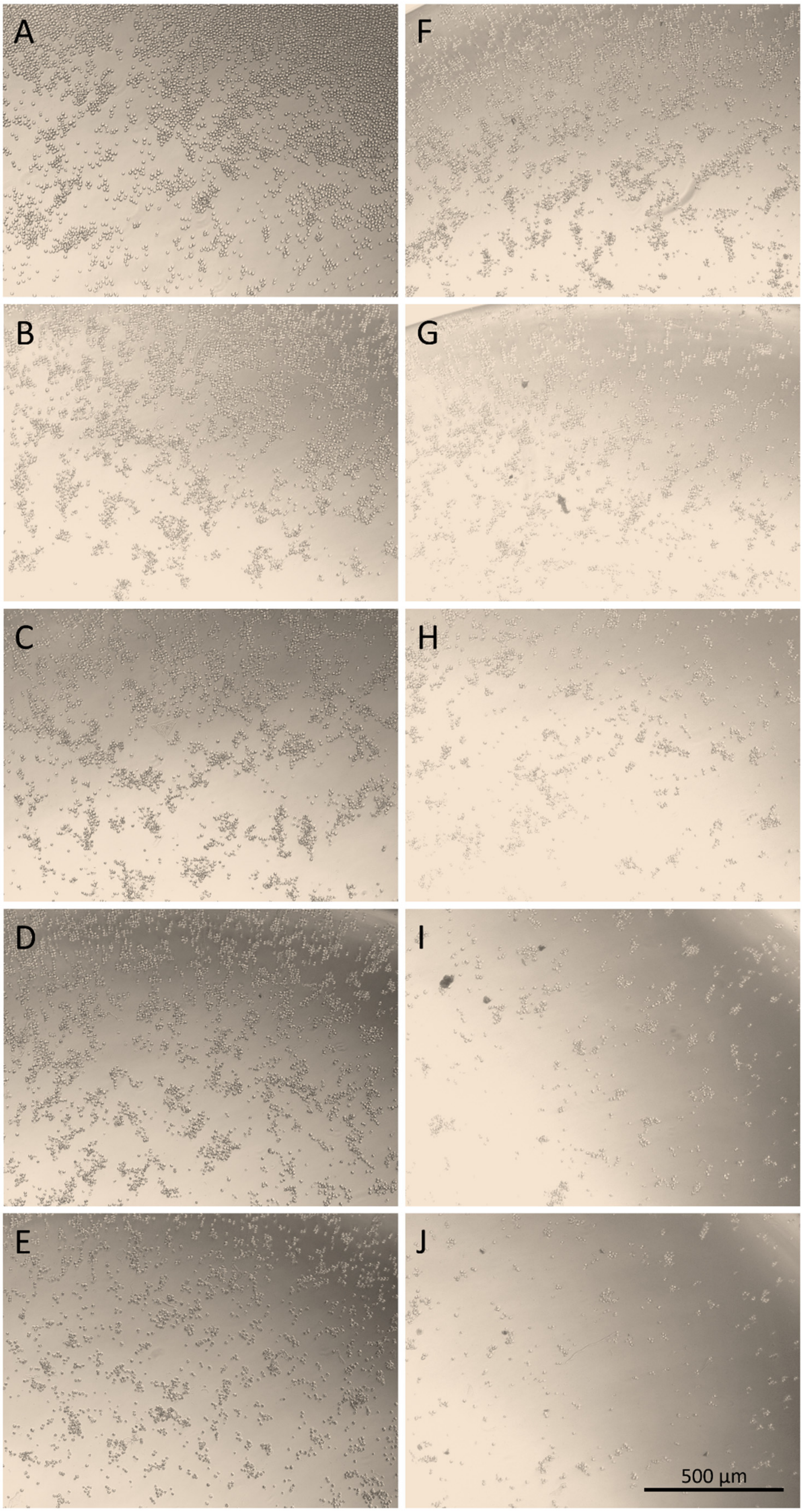

Figure S8. Bright field images of MV4-11 cells incubated for 24 hours with different GNP-MDS-PLU volumes: (A) control, 0 µl; (B) 2.5 µl; (C) 5 µl; (D) 10 µl; (E) 15 µl; (F) 20 µl; (G) 30 µl; (H) 50 µl; (I) 75 µl; (J) 100 µl.
